# The Genetic Diversity of Cranberry Crop Wild Relatives, *Vaccinium macrocarpon* Aiton and *V. oxycoccos* L., in the US, with Special Emphasis on National Forests

**DOI:** 10.3390/plants9111446

**Published:** 2020-10-26

**Authors:** Lorraine Rodriguez-Bonilla, Karen A. Williams, Fabian Rodríguez Bonilla, Daniel Matusinec, Andrew Maule, Kevin Coe, Eric Wiesman, Luis Diaz-Garcia, Juan Zalapa

**Affiliations:** 1 Department of Horticulture, University of Wisconsin, Madison, WI 53706, USA; rodriguezbon@wisc.edu (L.R.-B.); dmatusinec@wisc.edu (D.M.); maule2@wisc.edu (A.M.); coe3@wisc.edu (K.C.); 2 USDA-ARS, National Germplasm Resources Laboratory, Beltsville, MD 20705, USA; 3 Department of Plant and Environmental Sciences, Clemson University, Clemson, SC 29634, USA; fabianr@clemson.edu; 4 USDA-ARS, Vegetable Crops Research Unit, University of Wisconsin, Madison, WI 53706, USA; eric.wiesman@usda.gov; 5 Instituto Nacional de Investigaciones Forestales, Agrícolas y Pecuarias (INIFAP), Aguascalientes 34170, Mexico; diaz.antonio@inifap.gob.mx

**Keywords:** cranberry breeding, crop wild relatives, genetic diversity, genetic distance

## Abstract

Knowledge of the genetic diversity in populations of crop wild relatives (CWR) can inform effective strategies for their conservation and facilitate utilization to solve agricultural challenges. Two crop wild relatives of the cultivated cranberry are widely distributed in the US. We studied 21 populations of *Vaccinium macrocarpon* Aiton and 24 populations of *Vaccinium oxycoccos* L. across much of their native ranges in the US using 32 simple sequence repeat (SSR) markers. We observed high levels of heterozygosity for both species across populations with private alleles ranging from 0 to 26. For *V. macrocarpon*, we found a total of 613 alleles and high levels of heterozygosity (H_O_ = 0.99, H_T_ = 0.75). We also observed high numbers of alleles (881) and levels of heterozygosity (H_O_ = 0.71, H_T_ = 0.80) in *V. oxycoccos* (4x). Our genetic analyses confirmed the field identification of a native population of *V. macrocarpon* on the Okanogan-Wenatchee National Forest in the state of Washington, far outside the previously reported range for the species. Our results will help to inform efforts of the United States Department of Agriculture Agricultural Research Service (USDA-ARS) and the United States Forest Service (USFS) to conserve the most diverse and unique wild cranberry populations through ex situ preservation of germplasm and in situ conservation in designated sites on National Forests.

## 1. Introduction

Crop wild relatives (CWR), in addition to being important components of ecosystems, are critical sources of genetic variation for crop improvement programs. These wild relatives often display more genetic variability than their cultivated counterparts and their evolution in the wild is dynamic, driven by interactions with both biotic and abiotic stressors [1]. The use of these resources in breeding programs is increasing as molecular applications are used to identify and facilitate the introgression of traits from CWR into cultivated varieties [2]. To support current and future sustainable agriculture and food security, these resources must be conserved and made available using a complementary strategy involving both ex situ and in situ conservation. The use of estimators of genetic diversity, such as number of private alleles, heterozygosity, and population differentiation, can help inform conservation decisions, including the prioritization of populations for protection as in situ reserve [3].

The American cranberry (*Vaccinium macrocarpon* Aiton) is a diploid perennial species with a natural distribution ranging from Newfoundland to the southern Appalachian Mountains, and extending west to Minnesota [4,5]. It occurs on acid soils and peat of bogs, swamps, wet shores, headlands, and occasionally poorly drained upland meadows [4]. It is found growing with other species adapted to the conditions in these environments, including sphagnum mosses, other ericaceous shrubs, graminoids, insectivorous plants, and widely scattered coniferous trees.

The majority of the existing cranberry cultivars, upon which the cranberry industry is based, are derived from wild selections of *V. macrocarpon* known as the “Big Seven” made in the 19th and 20th centuries [6,7,8]. The value of the industry, currently around $3.5. billion, continues to grow with high demand for products made with cranberry, which has a high nutritional content and one of the highest antioxidant profiles found in fruits [9,10,11]. Breeding programs are actively searching for sources of desirable traits that can be incorporated into new cultivars to withstand the pressures of diseases and insects as well as environmental stresses related to climate change, which in cranberry has resulted in crop loss due to frost damage [8,12,13].

The small cranberry, *Vaccinium oxycoccos* L. (4x), is morphologically very similar to the American cranberry and grows in similar environments as a small, evergreen, perennial vine [4,14]. *Vaccinium oxycoccos* produces small over-wintering berries that have a flavor profile similar to cultivated cranberry, but exhibit a superior antioxidant profile and cold hardiness [11,15]. As a wild relative to the cultivated cranberry, *V. oxycoccos* has great potential to be used as a parent in interspecific crosses to transfer desirable traits. In particular, since *V. oxycoccos* has a circumboreal distribution and is adapted to growing in higher latitudes than *V. macrocarpon* [16], the species could be an excellent source of cold hardiness genes, as is the case for a wild relative of rice [17].

Molecular markers such as simple sequence repeats (SSR) have been identified for many plant species and successfully used to gain insights into the genetic diversity of CWR of several crops, including apple [18], wheat [19], sugarcane [20], and sweet potato [21]. In cranberry, SSR markers have been used to evaluate the genetic diversity of wild populations in Wisconsin and Minnesota [22,23] and have proven to be transferable across species in the genus *Vaccinium* [24], which will also facilitate interspecific breeding and fingerprinting. Results from genetic diversity analyses have provided sufficient information to aid in situ conservation efforts in several plant species, such as *Borderea chouardii* Gaussen and Heslot, an endangered plant in Spain [25], and wild potatoes in Argentina [26].

**Figure 1:**
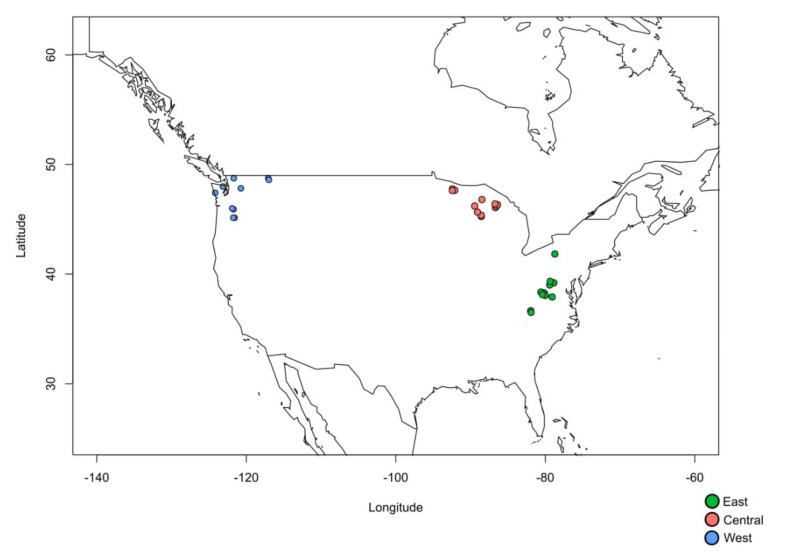
National forests and adjacent locations with populations of *Vaccinium macrocarpon* and *Vaccinium oxycoccos* included in the study.

Information on the genetic diversity of CWR necessary to inform the development of effective conservation strategies is limited. The occurrence of the wild cranberry species *Vaccinium macrocarpon* and *V. oxycoccos* on National Forests located throughout much of the ranges of the species, as well as collaborations between the United States Department of Agriculture Agricultural Research Service (USDA-ARS) and the United States Forest Service (USFS), and the University of Wisconsin, provided an opportunity to gain knowledge about the genetic diversity and population structure of these species (Figure 1, Table 1). These insights can help to prioritize conservation efforts for cranberry genetic resources in the US.

## 2. Results

### 2.1. Exploring the Genetic Relationships of Wild Cranberries

**Figure 2:**
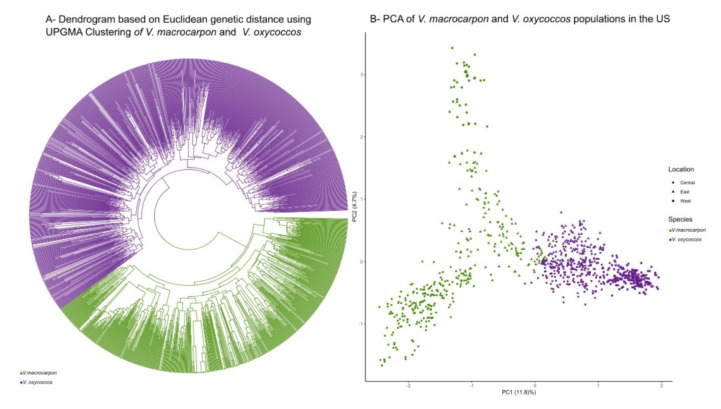
(**A**,**B**) Wild cranberry (*Vaccinium macrocarpon* and *Vaccinium oxycoccos*) species separation based on genetic distance.

The ranges of the two species in our study, *Vaccinium macrocarpon*, which is mostly restricted to North Central and Eastern North America, and *V. oxycoccos*, which has a circumboreal distribution, overlap in many areas in the US. Within these areas of sympatry, the species often co-exist on the same site, where they are sometimes difficult to differentiate (Figure 1, Table 1). To explore the genetic relationships both between and within species, we used a dendrogram based on Euclidean genetic distance with an unweighted pair group method with arithmetic mean (UPGMA) clustering method (Figure 2A). We found that both species separate clearly into two main groups, and all the field classifications made by USDA-ARS and USFS botanists were correct (Figure 2A). The UPGMA tree contained one branch with all the accessions identified in the field as *V. oxycoccos*, and another branch with all the *V. macrocarpon* individuals. To confirm our findings, we produced a principal component analysis (PCA) of all the populations, in which we also saw a clear separation between the two species (Figure 2B). In the PCA, we observed that the *V. macrocarpon* samples formed clearer spatial groupings, as seen in the separation of the eastern (Tennessee, North Carolina, Pennsylvania, and West Virginia), central (Wisconsin, Michigan, Minnesota), and western (Oregon, Washington, Idaho) regions, than those of *V. oxycoccos*. The one western population identified by USDA-ARS botanists as *V. macrocarpon* was at Site 34-OWFLBWA in the Okanogan-Wenatchee National Forest in Washington state, and its identity was corroborated by the genetic analysis (Figure 2B).

An overall comparison of genetic diversity estimators among all the individuals of the two species demonstrated high levels of heterozygosity. *Vaccinium oxycoccos* had the highest number of alleles with a total of 881 (n = 539) and a mean value of alleles per locus of 27.51. Also, *V. oxycoccos* had low levels of inbreeding, as demonstrated by the *G_IS_* value of 0.02, as well as a low fixation index (0.09). Overall, *V. macrocarpon* had a total of number of alleles of 613 (n = 388) and a mean value of 19.15 alleles per locus. *Vaccinium macrocarpon* also exhibited low levels of inbreeding (*G_IS_*- 0.95), but higher values of the fixation index (0.33) than *Vaccinium oxycoccos* (Table 2).

### 2.2. Genetic Diversity Estimators and Population Structure of Vaccinium macrocarpon

The mean number of alleles per population* ranged from 2.00 to 7.15 in *V. macrocarpon* (*values not shown for all populations due to low number of individuals). Also, we observed high levels of all genetic diversity estimators (H_O_, H_S_, H_T_), ranging from 0.21 to 1. Meanwhile, levels of inbreeding were very low, varying from −3.58 to −0.46 (Table 3). The majority of the populations exhibited high values of observed heterozygosity, but low levels of within population heterozygosity.

**Figure 3:**
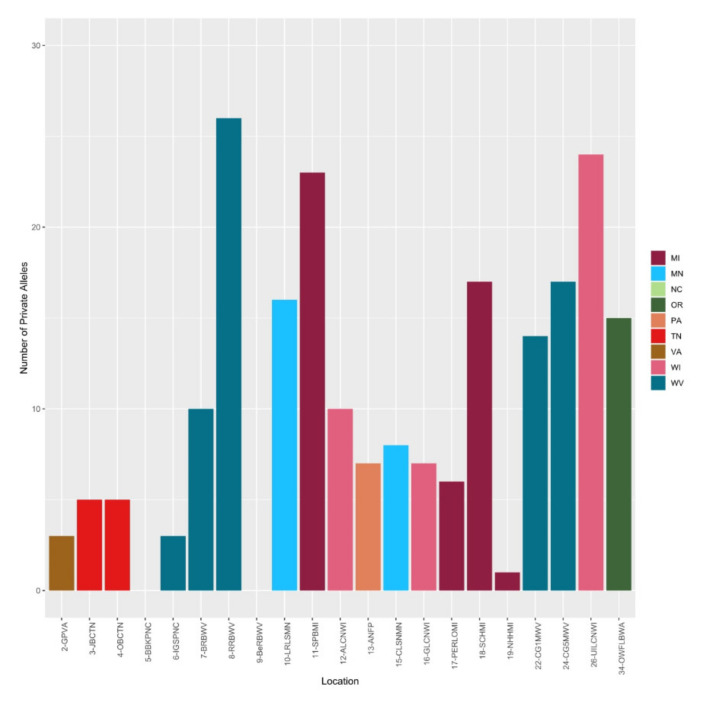
Number of private alleles per population of *Vaccinium macrocarpon*.

The number of private alleles per population of *V. macrocarpon* ranged from 0 to 26. The highest numbers of private alleles were found in populations at Red Run Bog in the Monongahela National Forest in West Virginia (26), Upper Island Lake in the Chequamegon-Nicolet National Forest in Wisconsin (24), Sand Point Bog in the Keweenaw Bay Indian Community in Michigan (23), and Cranberry Glades Site 5 in the Monongahela National Forest (17) (Figure 3).

**Figure 4:**
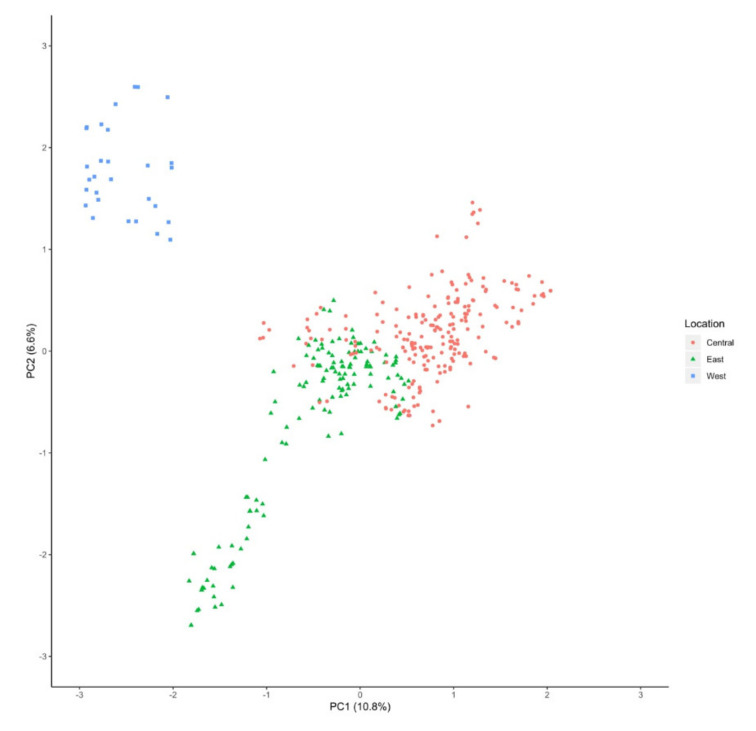
Principal component analysis (PCA) of *Vaccinium macrocarpon* populations.

A PCA was performed to understand the genetic relationships among *V. macrocarpon* populations (Figure 4). We observed a clear differentiation between the samples collected at Fish Lake Bog in the Okanogan-Wenatchee National Forest in Washington (34-OWFLBWA), which was genetically identified as *V. macrocarpon* in the Euclidean distance dendrogram, and samples from other populations. We also observed a separation between populations from central and eastern locations. There was a large spread among the eastern populations, which have historically been considered the most ancient populations of *V. macrocarpon* [27].

**Figure 5:**
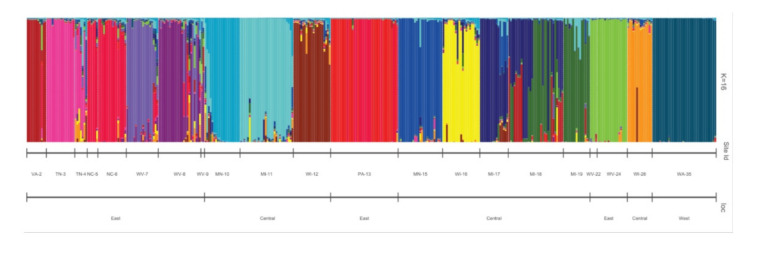
Structure analysis of *Vaccinium macrocarpon* populations (K = 16).

A structure analysis was performed to visualize any potential population groupings (Figure 5). Based on Evanno’s deltaK [28,29], we found that K = 16 was the most likely number of clusters, with the majority of these forming based on their geographical locations. Populations from West Virginia with Site IDs WV-7, WV-8, and WV-(22 and 24) formed distinct clusters, and we observed similar patterns for populations from Minnesota with Site IDs MN-10 and MN-15, from Wisconsin with Site IDs WI-12, WI-16, and WI-26, and from Michigan with Site IDs MI-11, MI-17, and MI-18. The population with Site ID MI-18 from the Hiawatha National Forest in Michigan is highly admixed and the population from Site ID WA-34 in the Okanogan-Wenatchee National Forest in Washington shows almost no admixture (Table 1, Figure 5).

### 2.3. Genetic Diversity Estimators and Population Structure of Vaccinium oxycoccos

The mean number of alleles per population for *V. oxycoccos* ranged from 2.71 to 11.3. The levels of heterozygosity for all three indexes (H_O_, H_S_, H_T_) ranged from 0.45 to 0.83, while the levels of inbreeding (*G_IS_*) ranged from −0.31 to 0.23 (Table 4). The populations with the highest diversity indices were located at the Pond East of Raven Lake (17-PERLOMI) in the Ottawa National Forest in Michigan and Glocke Lake (16-GLCNWI) in the Chequamegon-Nicolet National Forest in Wisconsin (Table 1, Table 4).

**Figure 6:**
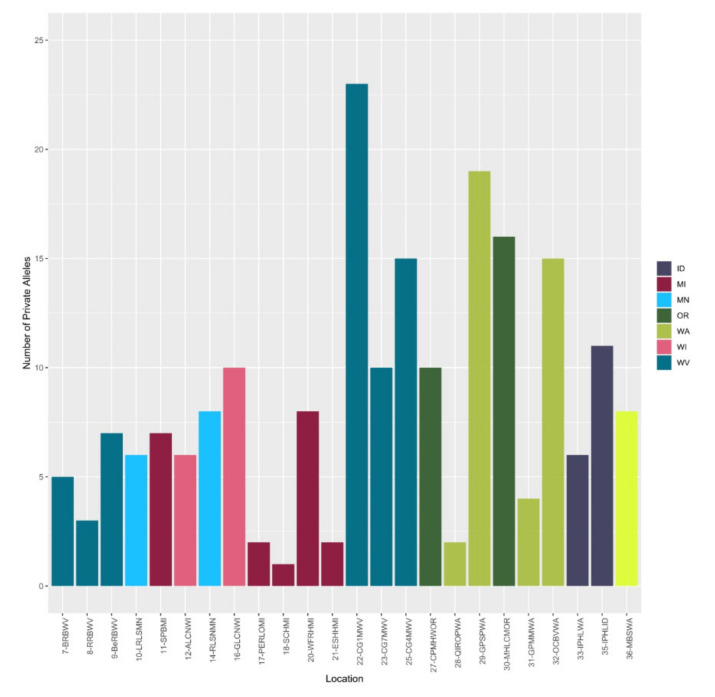
Number of private alleles per population of *Vaccinium oxycoccos*.

The analysis of private alleles per population of *V. oxycoccos* ranged from 1 to 23. The populations with the highest numbers of private alleles included Cranberry Glades site 1 in the Monongahela National Forest, West Virginia (23), South Prairie in the Gifford Pinchot National Forest, Washington (19) and Little Crater Meadow in Mount Hood National Forest, Oregon (16) (Figure 6).

**Figure 7:**
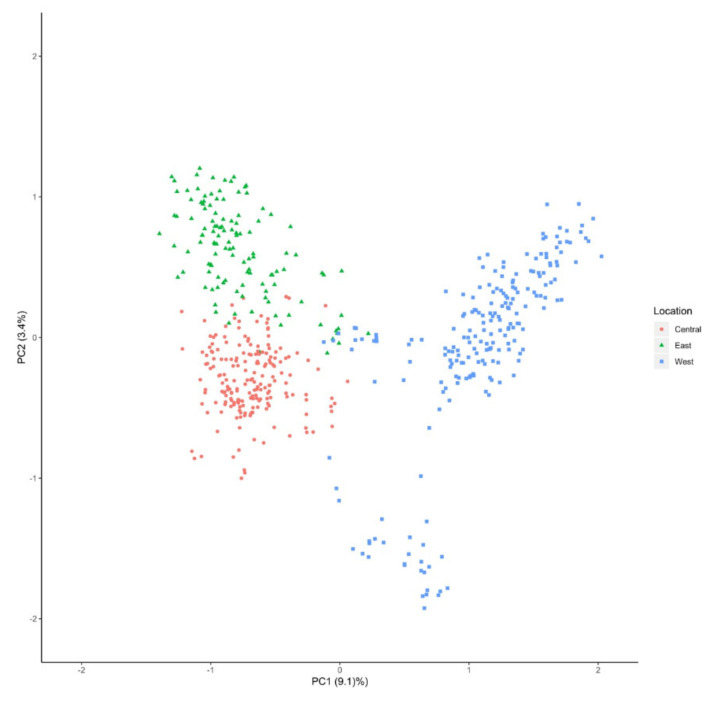
Principal component analysis (PCA) of *Vaccinium oxycoccos* populations.

A PCA was constructed to observe any groupings among *V. oxycoccos* samples (Figure 7). As in the *V. macrocarpon* PCA (Figure 4), we observed a clear geographical separation among the samples from western, central, and eastern locations. The distribution of the data in this PCA showed a closer association of the samples than the PCA for *V. macrocarpon*, suggesting a closer genetic relationship among *V. oxycoccos* individuals. 

**Figure 8:**
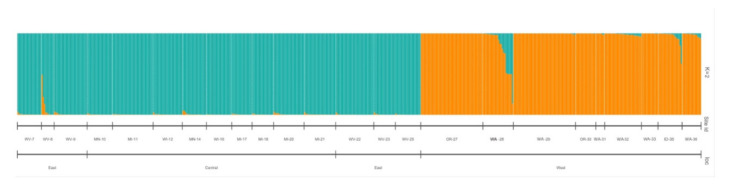
Structure analysis of *Vaccinium oxycoccos* populations (K = 2).

We performed a population structure analysis of the *V. oxycoccos* populations to better understand if any other grouping was occurring. In the structure analysis K = 2 was the most likely number of clusters for this species (Figure 8). One cluster was composed of all the populations sampled in the Eastern and Central US and the second cluster of the populations sampled in the Western US, with populations in the Quinault Indian Reservation (28-QIROP-WA) in Washington and the Idaho Panhandle National Forest (35-IPHLID) showing some levels of admixture. 

### 2.4. Analysis of Variable Regions in the Cranberry Organellar Genomes

**Figure 9:**
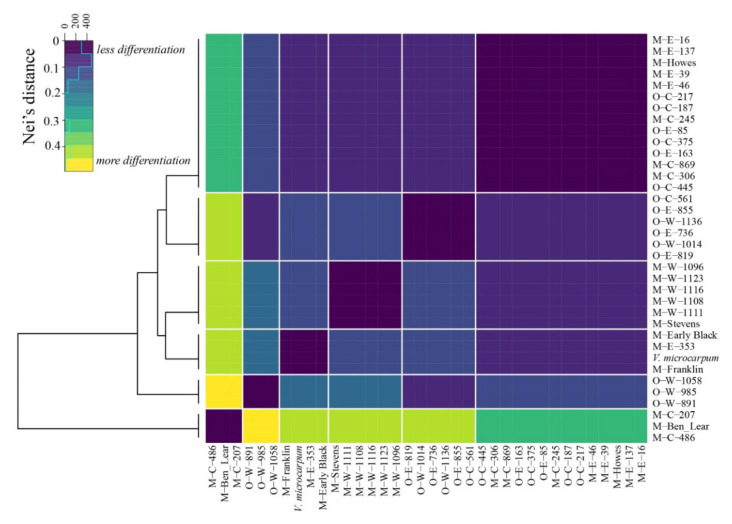
Genetic distance of *Vaccinium macrocarpon* cultivars/wild accessions (M), wild *Vaccinium oxycoccos* (O) and *Vaccinium microcarpum* from across the US (west = W, east = E, and central = C) based on organellar single nucleotide polymorphisms.

In order to understand genetic relationships between samples from different locations and cultivars (Table S1), we clustered the samples based on Nei’s genetic distance of six organellar genes and two INDELS that have previously shown to be the only variable regions in the organellar genomes of cranberry cultivars and its wild relative *V. microcarpum* [30,31]. The largest genetic distance of 1 was observed between *V. macrocarpon* and *V. oxycoccos* samples collected in Washington and *V. macrocarpon* samples collected in Wisconsin and Minnesota [M-C-486 (18-SCHMI), M-Ben-Lear, and M-C-207(10-LRLSMN)], with two exceptions being O-W-1014 (30-MHLCMOR) and O-W-1136 (36-MBSWA), which clustered with samples collected in the Eastern US as well as cultivars (Figure 9; Table S1). Additionally, when considering only samples collected in Wisconsin and Minnesota and the Eastern US, genetic distances ranged from 0 (meaning no genetic differentiation was observed) to 0.68. 

A PCA (Figure S1) analysis using SSRs identified consistent and clear separation between *V. macrocarpon* cultivars/native selections and the *V. macrocarpon* population in the Western US (Site 34-OWFLBWA in the Okanogan Wenatchee National Forest), suggesting that the western *V. macrocarpon* population is not genetically closely related to known cultivars/native selections.

## 3. Discussion

Wild relatives of many crops, including corn, sunflower, wheat, rice, potato, tomato, and banana have been the source of genetic variability and beneficial traits, such as disease resistance and abiotic stress tolerance, for crop improvement [32]. In cranberry, many issues that can severely limit production [33], such as frost risk [12] and cranberry fruit rot (*Physalospora vaccinii* (Shear) Arx and E. Müll.), can be addressed by traits found in wild relatives of cranberry. In order to confront emerging threats while increasing yield and producing excellent quality fruit, wild genetic resources of cranberry should be evaluated, safeguarded, and made available. These resources may hold the genetic variation needed to overcome current and future challenges to cranberry production.

### 3.1. Genetic Analysis Shows High Differentiation between Morphologically Similar Species

The ranges of *Vaccinium macrocarpon* and *V. oxycoccos* overlap in many areas [14], with the species growing together in some locations (Table 1). To better understand the relationships between these two species we studied how samples from wild populations grouped based on genetic distance and morphological identification. We constructed a dendrogram using Euclidean distance with UPGMA clustering (Figure 2A), in which we observed two main clusters, one that contained all *V. oxycoccos* individuals and one that encompassed all *V. macrocarpon* samples. To further corroborate our results, a PCA was developed in which we observed similar results for species separation, as well as some geographical clustering within species (Figure 2B). Clear separation between the two species has been previously observed using amplified fragment length polymorphism (AFLP) [34] and SSR markers [22]. Rodriguez-Bonilla et al. [23] also observed a clear differentiation between species collected in natural areas in the states of Wisconsin and Minnesota, demonstrating the ability of these markers to differentiate between closely related species. 

Genetic estimates for both species showed very high levels of heterozygosity (H_O_, H_T_, H_S_) (Table 2), which are comparable to those found by Zalapa et al. [22] and Rodriguez-Bonilla et al. [23], who found high levels of heterozygosity in wild populations in both Wisconsin and Minnesota. These results are supported by cranberry biology, which favors outcrossing through dichogamy and inbreeding fertility reduction [35]. Moreover, natural hybridization can result in new genetic combinations due to chance seedlings [36]. The high levels of genetic diversity present in *V. oxycoccos* could be related to the polyploid nature of this species, a correlation seen in other polyploid crop wild populations, including cassava and sweet potato [21,37].

When analyzing other parameters of population differentiation, we observed that *V. macrocarpon* exhibited a much higher fixation index (0.33) and population structure compared to *V. oxycoccos* (0.09) (Table 2), which appeared to be more panmictic and less differentiated. Many authors believe that some populations of *V. macrocarpon* are relics of the Pleistocene Ice Age and, therefore, more differentiation among populations is expected [27,35,38].

### 3.2. Insight into the Genetic Diversity of Wild Populations of Vacciniium macrocarpon

Our results demonstrate that wild populations of cranberry harbor greater diversity than previously observed in other studies using markers such as random amplified polymorphic DNAs (RAPDs) and allozymes [38,39,40]. Codominant markers such as SSRs have also provided great insights into the genetic diversity of several other species, including rice [41], lima beans [42], and sweet potato [21].

In our study, most populations of *V. macrocarpon* exhibited high levels of genetic diversity, low levels of inbreeding, and moderate levels of within-population heterozygosity (Table 3). Zalapa et al. [22], Schlautman et al. [43] and Rodriguez-Bonilla et al. [23] also found high levels of heterozygosity and low levels of inbreeding in wild populations found in Wisconsin and Minnesota. Other diploid species such as roses have also been found to be highly heterozygous [44]. In wild cranberry populations, the outcrossing preference and the production of chance seedlings in nature likely contributes to the preservation of high levels of genetic diversity [35].

Using PCA, we identified three main clusters: one containing the population in Washington (34-OWFLBWA), one containing samples from the Eastern US, and one composed of all the Central US samples (Figure 4). To further explore the structure of these populations, we performed a cluster analysis, for which K = 16 was our best K based on Evanno’s deltaK method [28] (Figure 5). The observed results were expected based on the results obtained from the fixation index (0.33). Structure results showed a more in-depth differentiation of populations than the one observed in the PCA. We identified several populations from the same states (West Virginia, Wisconsin, Michigan, and Minnesota) within the main cluster that clustered separately based on Structure (Figure 5). These results suggest that these locations, although still genetically similar, have undergone genetic differentiation, potentially a result of isolation by distance [45].

### 3.3. Wild Vaccinium macrocarpon from Washington: An Escape or a Unique Population?

*V. macrocarpon* is naturally distributed mostly in the Eastern US and Canada and has been reported to naturally occur only as far west as Minnesota, with wild populations in the west generally thought to be the result of human introductions from the east. In our study, a population in the Okanogan-Wenatchee National Forest in Washington (34-OWFLBWA), was identified through field visual inspection as *V. macrocarpon* [4], and the identity was confirmed by our genetic analyses. This population was genetically differentiated from cultivars and selections of *V. macrocarpon* from the eastern and central US (Figure S1). In our Euclidean genetic distance dendrogram this population clearly clustered away from *V. oxycoccos* (Figure 2A) and also exhibited a clear separation from other *V. macrocarpon* samples in the PCA (Figure 2B).

Historical records indicate that *Vaccinium macrocarpon* was introduced to the Western US in the late 1800s [6]. Anthony Chabot was the first to introduce this species to southwestern Washington. McFarlin (which eventually was used as the mother plant to develop Stevens, the most frequently cultivated variety in the US), a native selection from Massachusetts, was the first cultivar to be grown in Oregon and Washington. Because of this history we first theorized that the population in the Okanogan-Wenatchee National Forest (34–OWFLBWA) in Washington could be an escape from cultivation, even though there is no cranberry production east of the Cascades Mountain Range in the state. We performed a PCA with 11 SSR markers using known eastern cultivars and native selections from central and eastern locations, and our population from the Okanogan-Wenatchee National Forest. We observed a clear spatial separation between the cultivars and native selections and our wild western accession based on PC1. We also observed the expected separation between cultivars and native selections based on PC2. These results suggest that the population in Washington is in fact genetically distinct from known cranberry germplasm (Figure S1).

If the population from the Okanogan-Wenatchee National Forest in Washington (34–OWFLBWA) was indeed established by escape from cultivation, we would have expected founder and bottleneck effects to have produced low levels of genetic variation [46]. On the contrary, we observed high levels of genetic diversity based on H_o_ = 0.97 and low inbreeding with *G_IS_* = -1.00 (Table 3), which are not consistent with the escape of a cranberry cultivar (i.e., single genotype clone). Additionally, in the *V. macrocarpon* clustering and population structure analysis, this population consistently clustered separately from the rest of the populations, exhibiting very little admixture (Figure 5). Cranberry cultivation is very localized in Washington, and the Okanogan-Wenatchee population is isolated and not near any commercial or cultivated marshes. Additionally, this population was well differentiated with vast diversity and private alleles (15) (Figure 3). Therefore, based on our data, it is not likely that the Okanogan-Wenatchee National Forest population was derived from cultivated cranberries originating in the Eastern or Central US.

### 3.4. Wild Cranberry Populations from Washington Could Help Unravel a Complicated Evolutionary History

Previous work by Diaz-Garcia et al. [31] observed that the cultivar Stevens (which is a cross of eastern and central native cranberry selections) shares almost identical organellar genomes as a *V. microcarpum* individual collected in the region of the ice-free corridor (IFC) in Alaska. One theory that may help explain these findings is that *V. microcarpum* from Alaska is the progenitor of the cultivated cranberry. According to this theory, *V. microcarpum* expanded its range through the North American IFC and then into the deglaciating parts of the Northeast US. This expansion possibly involved human, wildlife, or even water-mediated movement of plants and seeds through the IFC [47]. Based on these findings, and to further understand the relationship between population 34–OWFLBWA and other cranberries from both species, wild and cultivated, we sequenced eight regions that have shown to be the only organellar cranberry variable regions, two of which are plastid single nucleotide polymorphism (SNP) differences with *V. microcarpum* [30,31]. We used 30 representative samples from our wild collections in National Forests for both *V. macrocarpon* and *V. oxycoccos* from across the US from the current study plus five cranberry cultivars and a *V. microcarpum* accession [31] (Plant Materials are listed in Table S1). Since our wild collections did not include samples from the Northeastern US where the majority of the escaped *V. macrocarpon* populations now found in the Western US are thought to have originated, we included in our sequence analysis three popular eastern cultivars [wild selections Howes and Early Black (two of the “Big Seven” cultivars)] and Franklin (Early Black x Howes) plus a central cultivar Ben Lear (wild selection), and Stevens (Potters Favorite x McFarlin, both wild selections) and a *V. microcarpum* accession from Alaska (Table S1). 

We observed that samples from the population from the Okanogan-Wenatchee National Forest shared all analyzed SNPs and INDELS with Stevens, resulting in a genetic distance of 0 between samples of these groups. This result is consistent with the Stevens and *V. microcarpum* organellar study of Diaz-Garcia et al. [31], which also found near identical organellar genomes in the two species. Therefore, our study supports the idea that the Okanogan-Wenatchee National Forest population could also be a descendant of populations that moved through the IFC, and also the result of the incomplete lineage sorting that has been previously identified between *V. macrocarpon* and *V. microcarpum* [31]. Based on the location of this population, there is a possibility that it was introduced by humans or migratory wildlife through the IFC and changes in climate conditions prevented the expansion of these populations east, since the Fish Lake Bog area is bordered on the west by the Cascade Range and on the east by semi-desert.

These results suggest that this is the first genetically supported report of a unique native *V. macrocarpon* population west of Minnesota in the US. The existence of this *V. macrocarpon* population suggests that there could be other unique populations in the western bogs of Oregon, Washington, and Idaho. If the ancestors of *V. macrocarpon* originated from northern areas such as Alaska and spread through the North American IFC, the range of *V. macrocarpon* could have been widespread at one point. As the glaciers receded and environmental changes ensued, making much of the Western US arid and uninhabitable for cranberry populations, the Eastern and Central US and Canada might have been the only remaining hospitable habitats.

Regardless of origin, our findings indicate that *V. macrocarpon* populations are more widespread than previously believed and could be present in other western locations of the US and Canada, and at higher latitudes than previously believed. However, a more extensive survey, as well as morphological and genomic analyses of this population and other *V. microcarpum* accessions, is necessary to elucidate the evolution and migration of both species.

### 3.5. Insight into the Genetic Diversity of Wild Populations of Vaccinium oxycoccos

The *V. oxycoccos* populations in our study exhibited high levels of heterozygosity and alleles per population, as well as low levels of inbreeding (Table 4). When compared to *V. macrocarpon*, *V. oxycoccos* had higher levels of within-population heterozygosity and total heterozygosity (Table 4). The PCA obtained showed good separation among samples from the different geographical areas, although the grouping was closer than for *V. macrocarpon* (Figure 7). Similar results were obtained in a study of European *V. oxycoccos*, in which great genetic variability among two populations was observed [48]. Studies of wild populations of *V. oxycoccos* in Wisconsin and Minnesota also observed comparable levels of heterozygosity, ranging from 0.7 to 1, and low levels of inbreeding [23].

To better understand the population structure present in *V. oxycoccos*, we performed a Structure analysis with K = 2 based on Evanno’s deltaK [28] (Figure 8). This analysis showed a clear geographical separation between the eastern/central populations, and western populations, but further differentiation was not observed as expected from the observed fixation index (0.09). A similar structure has been shown in switchgrass ecotypes from different regions clustered based on geographical distribution [49]. We observed some levels of admixture in individuals from a population in the Idaho Panhandle National Forest (35-IPHLID). These results could be due to human–plant exchange or migrating birds spreading seeds west or east. 

### 3.6. Understanding Genetic Variation in Cranberry and Its Implications for Conservation

Our main goal was to understand the genetic variation of wild cranberry species in National Forests to provide the USDA-ARS and US Forest Service with the genetic information needed to plan conservation actions for these important crop wild relatives. We looked at three diversity and uniqueness indicators: private alleles, heterozygosity, and population structure. Private alleles are those found in one population among a group of populations, and are a great measurement of uniqueness [50].

We observed that for *Vaccinium macrocarpon*, the populations on National Forests with the highest number of private alleles were Red Run Bog in Monongahela National Forest in West Virginia (26), Upper Island Lake in the Chequamegon-Nicolet National Forest in Wisconsin (24), and Cranberry Glades site 5 in the Monongahela National Forest in West Virginia (17) (Figure 3). Another population not in a National Forest (Sand Point Bog in the Keweenaw Bay Indian Community) also had a high number of private alleles. For *V. oxycoccos*, the populations with the highest number of private alleles were Cranberry Glades site 1 in the Monongahela National Forest, West Virginia (23), South Prairie in the Gifford Pinchot National Forest, Washington (19) and Little Crater Meadow in the Mount Hood National Forest, Oregon (16) (Figure 6). These populations also demonstrated high levels of genetic variation based on their heterozygosity values (Table 3, Table 4). The combination of these population genetic parameters, as well as the population structure, provides us with insights about which populations have high or unique genetic diversity and should be considered when designating in situ genetic reserves for conservation of wild cranberry in National Forests.

## 4. Materials and Methods

### 4.1. Plant Materials

Plant materials were collected by ARS and USFS botanists and technicians (Figure 1). In order to locate populations of *Vaccinium macrocarpon* and *V. oxycoccos*, online herbarium and germplasm records in the USDA National Plant Germplasm System (GRIN-Global), the Global Biodiversity Information Facility, the Global Crop Wild Relative Occurrence Database, the Genesys plant genetic resources portal, the Botanic Gardens Conservation International PlantSearch database, and the Consortium of Midwest Herbaria were searched for known populations on National Forests throughout the US. Scientific staff at the National Forests provided additional information on locations of populations. Collections from two other sites located near National Forests were also included in the analysis. The collection sites ranged from a small seep (14 m2) supporting the population at the southernmost latitude (5-BBKPNC) to large bogs and fens covering hundreds of thousands of square meters. The elevation of populations ranged from 183 m (11-SPBMI) to 1773 m (5-BBKPNC) for *V. macrocarpon* and from 114 m (28-QIROPWA) to 1196 m (9-BeRBWV) for *V. oxycoccos*. In the Eastern US, *V. macrocarpon* and *V. oxycoccos* were often found growing together on the same site. In the Western US, all populations were *V. oxycoccos*, except one that was determined to be *V. macrocarpon*.

Multiple samples of leaf tissue were collected at each site. Each sample was an upright (i.e., reproductive branch) or a runner (i.e., vegetative branch) containing at least 20 leaves from a single plant. When found together at the same site, *V. macrocarpon* and *V. oxycoccos* were sampled separately. Following the technique utilized by Zalapa et al. [22], samples within each species were collected at least 5 m apart to reduce the likelihood of collecting the same clone more than once. As much as possible of the entire population was covered by taking samples in a grid pattern. Latitude and longitude were determined for each sample and recorded on the paper coin envelope holding the sample. All samples for each species at a site were placed in a large paper envelope.

Each collection site was documented with location, physical characteristics, associated species, and descriptions of the populations and plants being sampled (available upon request). Latitude, longitude, and altitude were recorded using a Garmin GPSMAP 64st or other global positioning unit and/or altimeter. The geodetic datum WGS 84 was used.

Herbarium vouchers were deposited in the USDA National Arboretum in Washington, D.C. and the USDA National Germplasm Repository in Corvallis, Oregon.

### 4.2. DNA Extraction

Plant materials were provided to the USDA-ARS, Cranberry Genetics and Genomics Lab at the University of Wisconsin-Madison, for genetic analysis. Genomic DNA extractions for sequencing and diversity analysis were performed using leaf tissue per a CTAB method [51] modified in our laboratory to isolate high-quality, clean DNA. A total of 10–20 mg of frozen tissue were hand-ground using liquid nitrogen and then transferred to a 2.0 mL tube. 700 μL of 2% CTAB extraction buffer (20 mM EDTA, 0.1 M Tris-HCl pH 8.0, 1.4 M NaCl, 2% CTAB) was added to the tube and then mixed. The solution was then incubated at 65 °C for 45 min. After incubation, 400 μL of a chloroform-isoamyl alcohol (24:1) solution was added to the tubes and further mixed gently by inversion. Samples were then centrifuged for 5 min at 14,000 rpm; 500 μL of the supernatant was then transferred to a fresh 1.5 mL tube containing 50 μL of 10% CTAB buffer and then mixed. We added 750 μL of cold isopropanol (100%), and then samples were incubated from 2 to 48 h at −20 °C. Samples were then centrifuged at 14,000 rpm for 20 min. After centrifugation, the supernatant was discarded, and the resulting pellet air-dried for 5 min. The pellet was then washed with 700 μL of cold 70% ethanol, vortexed, and centrifuged at 14,000 rpm for 4 min. The ethanol was discarded and the pellet air-dried for approximately 24 h. The DNA was then re-suspended in 100 μL TE 10:1 buffer (10 mM Tris-HCl pH 8.0, 1 mM EDTA pH 8.0) plus 5 μL of ribonuclease (RNAse 10 mg mL−1) in each tube, and was incubated at 37 °C for 2 h prior to storage at − 20 °C.

### 4.3. Simple Sequence Repeat (SSR) Markers, Polymerase Chain Reaction (PCR) Conditions and Analysis of Diversity

A total of 32 markers previously designed and assessed for transferability between *V. macrocarpon* and *V. oxycoccos* were used in this study [7,24,52,53] (Table S1). Markers proven to have a consistently clear pattern of amplification across species were chosen, and at least two markers per cranberry linkage group previously known to exhibit Mendelian segregation patterns were selected for this study. PCR protocols used in this study were as described by Rodriguez-Bonilla et al. [23].

For fragment analysis, a total of 25 μL f PCR product and 1500 μL of formamide (Hi-Di Formamide from Life Technologies) were added per plate of 96 wells. The poolplexed mixture was sent to the University of Wisconsin-Madison Biotechnology Center DNA sequencing facility for fragment analysis using an ABI 3730 fluorescent sequencer (Applied Biosystems, Foster City, CA, USA). Allele genotyping was performed using GeneMarker v2.63 (SoftGenetics LLC, State College, PA, USA).

The allelic information obtained from genotyping was formatted as a GenAlEx [54] input file. This file was then converted to a geneclone object to run in the R statistical software [55] package Population Genetics in R (poppr) [56] to estimate the observed number of private alleles (PA). Calculations of genetic diversity statistics, such as number of alleles, H_O_, G’st(Nei), and *G_IS_*, were obtained from the software GenoDive [57]. PCA was performed converting a geneclone matrix into a genind object in adegenet [58]. Missing data (NA) values were replaced with mean allele frequencies. To visualize the PCAs, the packages ggplot2 and factoextra were used [59,60]. For the dendrograms, the R stats package [55] was used to obtain Euclidean genetic distance and clustering. The dendrograms were then visualized using the packages dendextend [61] and circlize [62]. Population structure analysis was performed using the Structure 2.3.4 with K’s 1 to 36, 150,000 MCMC (Markov Chain Monte Carlo), and 50,000 burn-in period [63]. The estimations of deltaK were obtained with Structure Harvester [28,29]. Data obtained from Structure was processed and visualized using the R package pophelper 2.2.9 [64]. To geographically visualize our forest locations we used the package maps [65].

### 4.4. Analysis of Organelle Sequence Data and Genetic Relatedness Analysis

To explore organellar genetic relatedness among populations from different locations in the US, we compared samples from the current study (*n* = 30: 15 each for *V. macrocarpon* and *V. microcarpum*) to cultivated cranberry samples (n = 5) and *V. microcarpum* samples (n = 1). Plant materials used in this analysis are listed in Table S1. Primers were designed to amplify eight previously observed variable regions in the plastid and mitochondrial genomes of *V. macrocarpon* cultivars and *V. microcarpum* as identified by Diaz-Garcia et al. [31] and Fajardo et al. [30] using the Geneious Prime® 2020.0.3 software. We sequenced six regions that have been shown to be the only organellar variable regions (four regions containing SNPs and two INDELs (insertion/deletion)) observed between the *V. macrocarpon* cultivars Ben Lear, HyRed, and Stevens [30]. We also included two other regions containing SNPs found between the organelles of cultivar Stevens and *V. microcarpum* [31].

PCR Reactions for Sanger sequencing contained 10.5 µL of 1x Jumpstart RedTaq Ready Mix (Sigma, St. Louis, MO, USA), 2.0 ul of 50 ng/ul DNA, 1 µL of 5 µM forward primer, 1 µL of 5 µM reverse primer. Thermocycling conditions consisted of 94 °C for 3 min, [94 °C 15s, 55 °C for 1 min, 72 °C for 2 min] 33, 72 °C for 30 min, 4 ∞. PCR products were submitted to the University of Wisconsin-Madison Biotechnology Center for Sanger sequencing both ways (forward and reverse).

The forward and reverse sequences obtained from Sanger Sequencing were aligned using a pairwise alignment, using the Geneious Alignment tool (global alignment with free end gaps). Aligned sequences were then trimmed and a consensus sequence was created in order to perform a multiple alignment of all individuals based on similarity using the Clustal Omega tool found on the Geneious Prime® 2020.0.3 software. Sequences were then batch exported in FASTA format for genetic distance analysis.

Sanger sequence reads for the six organellar genes were merged into a single FASTA file for each sample using the R package Biostrings v2.50.2 [66]. Sequences were then converted to a genlight object using adegenet, and Nei’s genetic distance [67] between each sample was calculated using Stampp [68]. The resulting pairwise genetic distance matrix was then plotted in ggmap [69] to visualize relationships between samples.

## 5. Conclusions

Our study elucidated the genetic diversity and population structure of wild populations of two species of wild cranberry located mainly in National Forests. The populations are distributed across the US and exhibit unique allelic combinations that could be useful for breeding, especially since cranberry is a relatively new crop that has not been the subject of much artificial selection. These results will contribute to decisions on which populations should be prioritized for conservation action, including site management and collection of germplasm, by the USDA Forest Service and Agricultural Research Service. We were also able to identify a unique population of *V. macrocarpon* outside its native range, which could be useful in better understanding the evolution of this complex section of the genus *Vaccinium*.

## Figures and Tables

**Table 1:** Sample Collection Locations for *Vaccinium macrocarpon* and *Vaccinium oxycoccos*.

Site ID	Species	Sample IDs	State	National Forest/ Administrative Area	Location	Latitude	Longitude	Regional Group
2-GPVA (VA-2) *	*V. macrocarpon*	VAC2013 (4–23)	Virginia	George Washington National Forest	Green Pond	37.94087	−79.0526	East
3-JBCTN (TN-3) *	*V. macrocarpon*	VAC2013 (25–40)	Tennessee	Cherokee National Forest	John’s Bog	36.52908	−81.964	East
4-OBCTN (TN-4) *	*V. macrocarpon*	VAC2013 (41–47)	Tennessee	Cherokee National Forest	Osborne Bog	36.48826	−81.9652	East
5-BBKPNC (NC-5) *	*V. macrocarpon*	VAC2013 (48–53)	North Carolina	Pisgah National Forest	Black Balsam Knob	35.32943	−82.8782	East
6-IGSPNC (NC-6) *	*V. macrocarpon*	VAC2013 (54–69)	North Carolina	Pisgah National Forest	Investor Gap Seep	35.34345	−82.8697	East
7-BRBWV (WV-7) *	*V. macrocarpon* *V. oxycoccos*	WV2014-2 (1–18) WV2014-1 (1–19)	West Virginia	Monongahela National Forest	Big Run Bog	39.11805	−79.5845	East
8-RRBWV (WV-8) *	*V. macrocarpon* *V. oxycoccos*	WV2014-4 (1–24) WV2014-3 (1–10)	West Virginia	Monongahela National Forest	Red Run Bog	39.07227	−79.4784	East
9-BeRBWV (WV-9) *	*V. macrocarpon* *V. oxycoccos*	WV2014-6 (1–2) WV2014-5 (1–26)	West Virginia	Monongahela National Forest	Bear Rocks Bog	39.06535	−79.3051	East
10-LRLSMN (MN-10) *	*V. macrocarpon* *V. oxycoccos*	VAC2015-M (165–184) VAC2015-O (145–164)	Minnesota	Superior National Forest	Little Rice Lake	47.70847	−92.4411	Central
11-SPBMI (MI-11) *	*V. macrocarpon* *V. oxycoccos*	VAC2015-M (220–254) VAC2015-O (185–219)	Michigan	Keweenaw Bay Indian Community	Sand Point Bog	46.79143	−88.4669	Central
12-ALCNWI (WI-12) *	*V. macrocarpon* *V. oxycoccos*	VAC2015-M (278–298) VAC2015-O (255–277)	Wisconsin	Chequamegon-Nicolet National Forest	Atkins Lake	45.64951	−89.0397	Central
13-ANFPA (PA-13) *	*V. macrocarpon*	VAC2015-M (299–336)	Pennsylvania	Allegheny National Forest	Along both sides of FR320, approximately 0.2 miles south of junction with FR455	41.81581	−78.7341	East
14-RLSNMN (MN-14) *	*V. oxycoccos*	VAC2015-O (337–356)	Minnesota	Superior National Forest	Rice Lake	47.56709	−92.3689	Central
15-CLSNMN (MN-15) *	*V. macrocarpon*	VAC2015-M (357–381)	Minnesota	Superior National Forest	Cranberry Lake	47.51161	−92.0166	Central
16-GLCNWI (WI-16) *	*V. macrocarpon* *V. oxycoccos*	VAC2015-M (382–402) VAC2015-O (403–422)	Wisconsin	Chequamegon-Nicolet National Forest	Glocke Lake	45.33362	−88.5702	Central
17-PERLOMI (MI-17) *	*V. macrocarpon* *V. oxycoccos*	USFS-ONF-2015-1-1, USFS-ONF-2015- 2 (1–16) USFS-ONF-2015-2 (1o–160)	Michigan	Ottawa National Forest	Pond east of Raven Lake	46.26263	−89.21718	Central
18-SCHMI (MI-18) *	*V. macrocarpon* *V. oxycoccos*	USFS-HNF-2015-1 (1–8, 12–17, 29–35, 38–47) USFS-HNF-2015-1 (9–11, 18–28, 36–37, 48)	Michigan	Hiawatha National Forest	South side FR2268 to east of H94, 0.8 miles south of Stutts Creek crossing	46.29175	−86.455	Central
19-NHHMI (MI-19) *	*V. macrocarpon*	USFS-HNF-2015-2 (1– 16)	Michigan	Hiawatha National Forest	North of Haywire Trail, unmarked two track West of Highway 94	46.29177	−86.454	Central
20-WFRHMI (MI-20) *	*V. oxycoccos*	USFS-HNF-2015-3 (1–24)	Michigan	Hiawatha National Forest	West Side of FR13, north of FR2447	46.1807	−86.4242	Central
21-ESHHMI (MI-21) *	*V. oxycoccos*	USFS-HNF-2015-4 (1–24)	Michigan	Hiawatha National Forest	East Side of Highway 13, north of FR2020	46.06647	−86.6438	Central
22-CG1MWV (WV-22) *	*V. macrocarpon* *V. oxycoccos*	USFS-MNF-2015-1-(11–12), USFS-MNF-2015-1-13 (k-p) USFS-MNF-2015-1- (1–10), USFS-MNF-2015-1 13 (a-j), (USFS-MNF-2015-1 (14–22)	West Virginia	Monongahela National Forest	Cranberry Glades 1	38.19939	−80.272	East
23-CG7MWV (WV-23) *	*V. oxycoccos*	USFS-MNF-2015-7 (1–16)	West Virginia	Monongahela National Forest	Cranberry Glades 7	38.20603	−80.2773	East
24-CG5MWV (WV-24) *	*V. macrocarpon*	USFS-MNF-2015-5 (1–17)	West Virginia	Monongahela National Forest	Cranberry Glades 5	38.19943	−80.2654	East
25-CG4MWV (WV-25) *	*V. oxycoccos*	USFS-MNF-2015-4 (1–20)	West Virginia	Monongahela National Forest	Cranberry Glades 4	38.20012	−80.2651	East
26-UILCNWI (WV-26) *	*V. macrocarpon*	USFS-CNNF-2015-4 (1–17)	Wisconsin	Chequamegon-Nicolet National Forest	Upper Island Lake	45.25023	−88.5576	Central
27-CPMHWOR (OR-27) *	*V. oxycoccos*	USFS-MHNF-2017-1 (1–49)	Oregon	Mount Hood National Forest	Camas Prairie	45.1382	−121.566	West
28-QIROPWA (WA-28) *	*V. oxycoccos*	QIR-2017-1 (1–24)	Washington	Quinault Indian Reservation	Otook Prairie	47.41137	−124.155	West
29-GPSPWA (WA-29) *	*V. oxycoccos*	USFS-GPNF-2017-2 (1–49)	Washington	Gifford Pinchot National Forest	South Prairie	45.90969	−121.699	West
30-MHLCMOR (OR-30) *	*V. oxycoccos*	USFS-MHNF-2017-2 (1–16)	Oregon	Mount Hood National Forest	Little Crater Meadow	45.14545	−121.741	West
31-GPMMWA (WA-31) *	*V. oxycoccos*	USFS-GPNF-2017-1 (1–7)	Washington	Gifford Pinchot National Forest	McClellan Meadows	45.99633	−121.89	West
32-OCBVWA (WA-32) *	*V. oxycoccos*	USFS-OLNF-2017-1 (1–29)	Washington	Olympic National Forest	Cranberry Bog Botanical Area	47.98635	−123.114	West
33-IPHLWA (WA-33) *	*V. oxycoccos*	USFS-IPMF-2018-1 (1–13)	Washington	Idaho Panhandle National Forest	Huff Lake	48.74059	−117.063	West
34-OWFLBWA (WA-34) *	*V. macrocarpon*	USFS-OWNF-2018-1 (1–36)	Washington	Okanogan-Wenatchee	Fish Lake Bog	47.8253	−120.723	West
35-IPHLID (ID-35) *	*V. oxycoccos*	USFS-IPNF-2018-2 (1–19)	Idaho	Idaho Panhandle National Forest	Hager Lake	48.59713	−116.97	West
36-MBSWA (WA-36) *	*V. oxycoccos*	USFSMBSNF-2018-1 (1–15)	Washington	Mt. Baker-Snoqualmie	Morovitz Wetland Complex	48.74092	−121.674	West

* Shorthand site IDs re used in the population structure analysis (Figure 5; Figure 8).

**Table 2:** Genetic diversity statistics for *Vaccinium macrocarpon* and *Vaccinium oxycoccos*.

	*V. macrocarpon*	*V. oxycoccos*
*Total alleles*	613	881
*N * ^1^	388	539
*Num * ^2^	19.15	27.51
*Ho * ^3^	0.99	0.71
*Hs * ^4^	0.51	0.72
*Ht * ^5^	0.75	0.80
*Gis * ^6^	−0.95	0.02
*G’st*(Nei) ^7^	0.33	0.09

^1^ Number of samples, ^2^ Mean number of alleles, ^3^ Observed heterozygosity, ^4^ Heterozygosity within populations, ^5^ Total heterozygosity, ^6^ Inbreeding Coefficient, ^7^ Nei’s fixation index.

**Table 3:** Genetic diversity statistics per population of *Vaccinium macrocarpon*.

Site ID	N ^1^	Num ^2^	Ho ^3^	Hs ^4^	Ht ^5^	Gis ^6^
2-GPVA	11	3.25	1.00	0.43	0.43	−1.28
3-JBCTN	16	2.31	1.00	0.23	0.23	−3.20
4-OBCTN	7	2.40	1.00	0.33	0.33	−2.01
5-BBKPNC	6	2.00	1.00	0.21	0.21	−3.58
6-IGSPNC	15	2.20	1.00	0.33	0.33	−2.03
7-BRBWV	18	4.10	0.99	0.55	0.55	−0.79
8-RRBWV	24	5.73	0.99	0.66	0.66	−0.48
10-LRLSMN	20	4.46	1.00	0.55	0.55	−0.81
11-SPBMI	31	7.15	1.00	0.68	0.68	−0.46
12-ALCNWI	21	5.31	1.00	0.62	0.62	−0.60
13-ANFPA	38	3.45	1.00	0.52	0.52	−0.89
15-CLSNMN	25	4.64	1.00	0.60	0.60	−0.65
16-GLCNWI	21	5.37	1.00	0.60	0.60	−0.66
17-PERLOMI	17	4.75	1.00	0.51	0.51	−0.93
18-SCHMI	31	6.40	1.00	0.63	0.63	−0.56
19-NHHMI	15	3.96	1.00	0.51	0.51	−0.94
24-CG5MWV	16	5.34	0.99	0.62	0.62	−0.58
26-UILCNWI	14	6.06	1.00	0.66	0.66	−0.49
34-OWFLBWA	35	3.12	0.97	0.48	0.48	−1.00

^1^ Number of samples, ^2^ Mean number of alleles, ^3^ Observed heterozygosity, ^4^ Heterozygosity within populations, ^5^ Total heterozygosity, ^6^ Inbreeding coefficient.

**Table 4:** Genetic diversity statistics per population of *Vaccinium oxycoccos*.

Site ID	N ^1^	Num ^2^	Ho ^3^	Hs ^4^	Ht ^5^	Gis ^6^
7-BRBWV	19	6.43	0.75	0.73	0.73	−0.02
8-RRBWV	10	4.75	0.72	0.83	0.83	0.12
9-BeRBWV	26	8.12	0.64	0.74	0.74	0.12
10-LRLSMN	20	8.81	0.69	0.74	0.74	0.06
11-SPBMI	32	11.3	0.69	0.77	0.77	0.10
12-ALCNWI	23	11.0	0.70	0.77	0.77	0.09
14-RLSNMN	19	9.16	0.70	0.78	0.78	0.10
16-GLCNWI	20	9.81	0.79	0.79	0.79	−0.00
17-PERLOMI	16	9.06	0.80	0.77	0.77	−0.03
18-SCHMI	16	6.50	0.73	0.70	0.70	−0.03
20-WFRHMI	24	9.65	0.74	0.77	0.77	0.03
21-ESHHMI	25	9.65	0.75	0.76	0.76	0.01
22-CG1MWV	30	10.3	0.74	0.76	0.76	0.01
23-CG7MWV	16	8.78	0.77	0.76	0.76	−0.01
25-CG4MWV	20	9.93	0.75	0.77	0.77	0.02
27-CPMHWOR	49	7.25	0.68	0.65	0.65	−0.05
28-QIROPWA	24	4.66	0.45	0.59	0.59	0.23
29-GPSPWA	49	9.45	0.75	0.69	0.69	−0.08
30-MHLCMOR	16	6.25	0.70	0.66	0.66	−0.07
31-GPMMWA	7	5.06	0.76	0.69	0.69	−0.11
32-OCBVWA	29	9.54	0.71	0.68	0.68	−0.03
33-IPHLWA	13	2.71	0.67	0.51	0.51	−0.31
35-IPHLID	19	4.78	0.72	0.64	0.64	−0.11
36-MBSWA	15	4.96	0.71	0.65	0.65	−0.10

^1^ Number of samples, ^2^ Mean number of alleles, ^3^ Observed heterozygosity, ^4^ Heterozygosity within populations, ^5^ Total heterozygosity, ^6^ Inbreeding coefficient.

## Supplementary Materials

The following are available online at https://www.mdpi.com/2223-7747/9/11/1446/s1, Figure S1: PCA of population 34-OWFLBWA population and cranberry cultivars, Table S1: Additional data referring to SSR and organelle sequences and samples used in Figure 9.
