# Supplementary figures and images for: The Genetic Diversity of Cranberry Crop Wild Relatives, Vaccinium macrocarpon Aiton and V. oxycoccos L., in the US, with Special Emphasis on National Forests
Source: Plants (Basel). 2020 Oct 26;9(11):1446. doi: 10.3390/plants9111446 (PMC7716231; doi:10.3390/plants9111446)

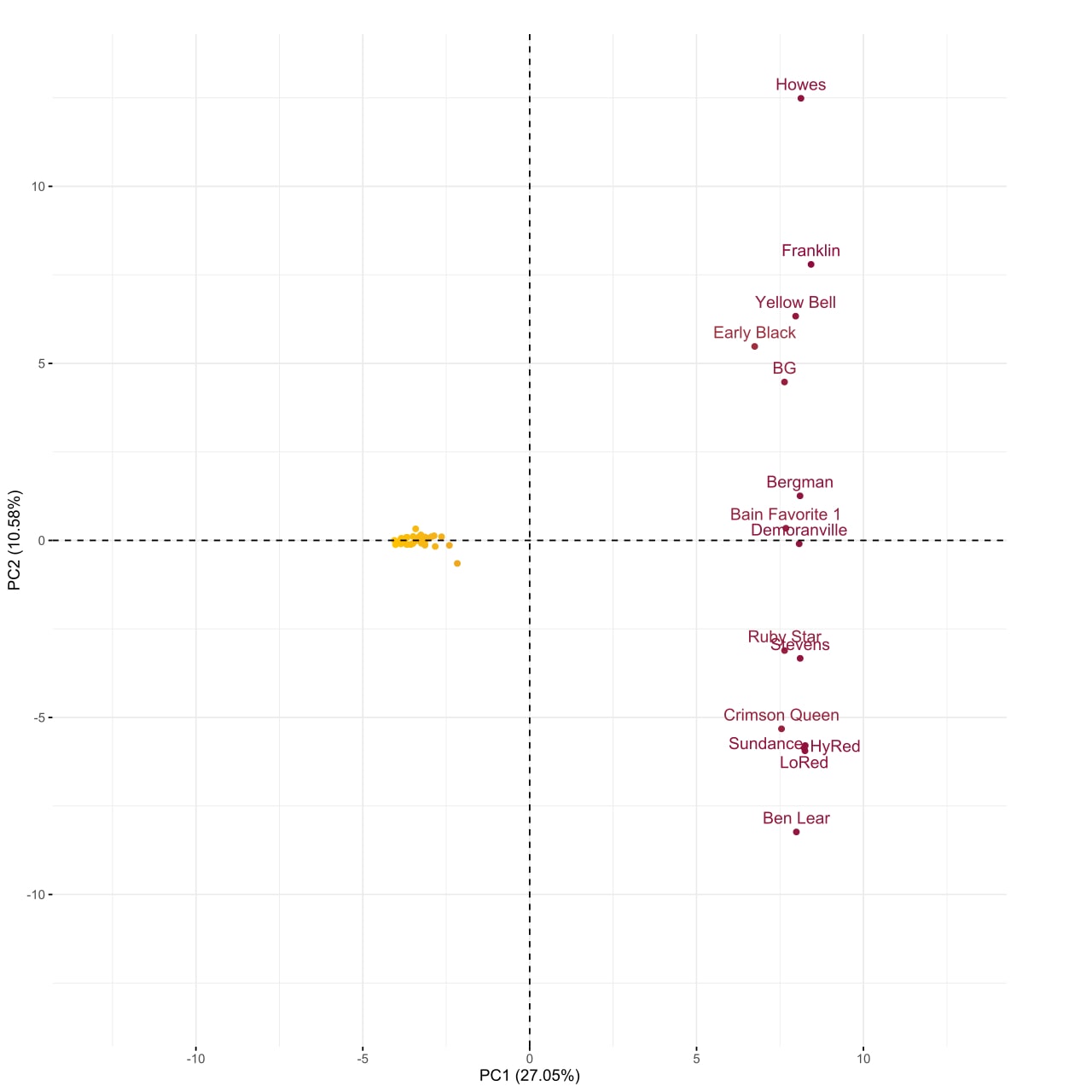

Supplement: Supplementary file 1 [file plants-09-01446-s001.zip › Fig 1 Supp Mat.jpg]
